# Efficacy of Smart Speaker–Based Metamemory Training in Older Adults: Case-Control Cohort Study

**DOI:** 10.2196/20177

**Published:** 2021-02-16

**Authors:** Jeongsim Kim, EunJi Shin, KyungHwa Han, Soowon Park, Jung Hae Youn, Guixiang Jin, Jun-Young Lee

**Affiliations:** 1 Department of Psychiatry Seoul National University College of Medicine and SMG-SNU Boramae Medical Center Seoul Republic of Korea; 2 Department of Clinical Counseling Psychology Cha University Seoul Republic of Korea; 3 Division of Teacher Education College of Liberal Arts and Interdisciplinary Studies, Kyonggi University Suwon Republic of Korea; 4 Social Value Innovation Center SK Telecom Seoul Republic of Korea

**Keywords:** smart speaker, cognitive training, cognitive decline

## Abstract

**Background:**

Metamemory training (MMT) is a useful training strategy for improving cognitive functioning in the older adult population. Despite the advantages, there are limitations imposed by location and time constraints.

**Objective:**

This study aimed to develop a smart speaker–based MMT program and evaluate the efficacy of the program in older adults without cognitive impairment.

**Methods:**

This study used a case-control cohort design. The smart speaker–based MMT program comprised 3 training sessions per day, 5 days a week, for 8 weeks. Each training session took approximately 15 minutes. This program was implemented using smart speakers, not human trainers. All participants completed the Mini-Mental State Examination, Subjective Memory Complaints Questionnaire, Verbal Learning Test, Digit Span Test, fluency tests, and a short-form version of the Geriatric Depression Scale before and after training.

**Results:**

A total of 60 subjects (29 in the MMT group and 31 in the control group) participated in the study. The training group showed significant increases in the delayed free recall, digit span forward, digit span backward, and fluency test scores compared with the control group.

**Conclusions:**

This study confirmed the efficacy of smart speaker–based MMT in older adults. Home-based smart speaker–based MMT is not limited with respect to location or constrained by space and may help older adults with subjective cognitive decline without requiring intervention by human professionals.

## Introduction

The gradual increase in the older adult population is leading to a growing problem with cognitive decline in the population. Many efforts have been made to address this concerning issue that is associated with older age.

The term “metamemory” was introduced by Flavell [1] and it refers to a type of metacognition, meaning the knowledge and awareness of an individual's own memory, including the contents and processes of their memory system [2]. Metamemory training (MMT) is a memory training program for the older population that is based on the metamemory concept, which consists of metaknowledge, meta-monitoring, and meta-judgment [3]. In the meta-knowledge component, MMT participants obtain information on how cognitive aging affects their memory abilities and how the brain operates memorizing processes. Throughout this part of the training, older people are educated about efficient strategies for dealing with cognitive aging. In the program’s meta-monitoring and meta-judgment components, participants develop the ability to monitor or judge their memory performance. Training programs help participants to understand memory and promote awareness of their own memory processing. This is accomplished through multimnemonic strategies (eg, storytelling, imagination, associations). MMT has been shown to have positive effects on everyday memory performance in older populations [4], as well as to improve memory and executive abilities in healthy older adults and adults with mild cognitive impairment [5-7]. MMT has also demonstrably enhanced the integrity of the white matter tract connecting the frontal and temporal cortices of the brain, which relate to the memory system [5].

Despite these advantages, there are some limitations to MMT. The first limitation involves the issues of accessibility and continuity. Many older adults have difficulty traveling to centers that run MMT programs. The second limitation relates to the insufficient number of experts available. Although the number of older adult populations experiencing cognitive decline continues to grow, the number of professionals nearby with expertise in the subject matter remains inadequate. It is difficult for older people to attend daily training sessions if they are living in rural areas where psychological experts are not readily available. Moreover, the active participation of people living in cities may be restricted by the usual business hours during which training is offered.

The emergence and integration of voice-user interfaces using artificial intelligence (AI) technology has led to the recent development of smart speakers. A smart speaker is a wireless and smart audio playback device that uses several types of connectivity for additional functions. They contain software that provides customized information or services to users while communicating with them by voice. Smart speakers have special features to enhance ease of use, connect to multiple types of audio sources, and provide additional functionality, and they are widely used in many countries. As international problems such as the COVID-19 situation have occurred, the “social distancing” culture has recently become the norm, and in such situations smart speakers that can be easily used at home and for various programs are becoming more and more popular [8].

Until now, there have been no smart speaker–based memory training programs, but there are some cognitive training tools that use computers or robots. Previous studies have reported that participants with high-functioning autism spectrum disorder who used computer-based virtual reality social training had improved social cognitive function [9,10]. In a previous study of participants with traumatic brain injury, improvements in memory abilities were reported following a virtual reality–based computer training program [11]. A recently published review of 11 studies on the effect of cognitive training with robots concluded that robot-based cognitive training in older adults with age-related cognitive decline could foster improved cognitive abilities [12]. None of these studies has overcome the above-mentioned limitations regarding location and time availability, such as requiring expert professional help or travel to a training center.

Although AI-based cognitive training has been found to be effective, the devices used for the training have some limitations. Users need to be able to use devices such as computers or robots, and many older adults are unfamiliar with their operation. These devices are expensive and challenging to deploy. On the other hand, smart speakers are affordable and offer a simple user interface that is verbally controlled. Therefore, older adults can use these devices with ease. Thus, smart speaker–based cognitive training may be a useful tool for overcoming the limitations of existing cognitive training programs.

Therefore, the MMT provided by the smart speaker method should be effective in improving cognitive function, especially in the area of fluency, as indicated by the results of previous studies. On that basis, we developed a smart speaker–based MMT program, trained older adults by having them participate in the program for 8 weeks without in-person training, and tested the program’s efficacy in a single-blind, case-control study.

## Methods

### Study Participants

**Figure 1:**
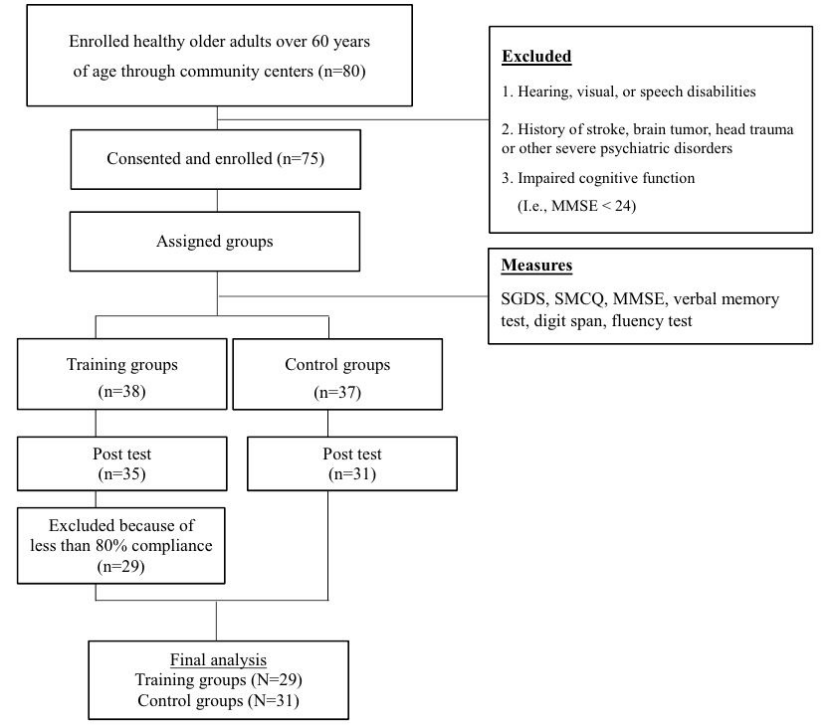
Study enrollment process. MMSE: Mini-Mental State Examination; SGDS: Short-Form Geriatric Depression Scale; SMCQ: Subjective Memory Complaints Questionnaire.

Figure 1 shows the patient enrollment process. Eighty people over the age of 60 years with normal cognitive function were recruited through community advertising and enrolled through three community centers. During the recruitment process, 5 potential participants were excluded because they met one of the following exclusion criteria: (1) hearing, visual, or speech disabilities; (2) history of stroke, brain tumor, head trauma, or severe psychiatric disorders; or (3) impaired cognitive functioning (ie, Mini-Mental State Examination score <24). The remaining 75 participants were assigned into either the training group (n=38) or the control group (n=37) using the convenience sampling method. The training group assignees were provided with SK Telecom’s smart speakers, while the control group assignees were not. Three participants in the training group and 6 participants in the control group were dropped from our analysis because they did not complete the poststudy evaluation. Thirty-five participants in the training group finished the smart speaker–based MMT and completed the pre/post cognitive tests. In the training group, we excluded 6 participants who had participation rates below 80%. Thirty-one participants in the control group completed the pre/post examinations. Finally, the analysis was conducted on 29 participants in the training group and 31 participants in the control group using per protocol analysis.

### Smart Speakers

The emergence and integration of voice interfaces using AI technology has led to the recent development of smart speakers. Smart speakers contain software that provides customized information or services to users while communicating with them by voice. The devices receive requests from users in the form of voice, text, or other communication forms and then performs the requested tasks.

As AI technology is at the core of smart speakers, the spoken language dialogue system (SLDS) proposed by Bertrand et al [13] is most similar to the system used in this study. SLDS functions include (1) speech recognition (including language understanding), (2) speech synthesis, and (3) dialogue management; all are similar to the current smart speaker system structure. Speech recognition technology refers to technology that receives human speech and converts it into text. This type of human–machine interaction technology can control various devices and services using human speech [14]. Speech synthesis mechanically produces human speech using a computer and is an active research focus due to the development of automation technology using deep neural networks. Recent synthesis technology that enables speech to reflect a specific tone has been developed to a level that makes various types of technical utilization possible, such as emotional expression [15]. Dialogue management receives text sentences from recognized human speech, understands the speaker’s intentions, and responds appropriately. Goal-oriented dialogue processing technology, which understands speech and maintains conversation on a related topic to accomplish a specific purpose or task, is used to implement smart speaker dialogue.

Based on voice recognition technology, smart speakers have voice interactions that can extract meaning from human voices, which leads to understanding between humans and machines. We call this a voice-user interface [16]. This voice interaction consists of voice recognition, voice synthesis, and dialogue management technologies. A system that provides voice interaction based on these technologies is called a conversational agent. Beyond their use as an interface, voice interactions with smart speakers are the key interaction determining the user’s main service experience. From the user's perspective, this is the most natural method of human interaction with machines.

### NUGU: The Smart Speaker Platform

This study was conducted using NUGU (SK Telecom [17]), a smart speaker platform that is widely used in Korea. According to a survey by SK telecom, there are currently about 1 million consumers with NUGU speakers. In August 2019, 670 million individuals used a NUGU speaker at least once a month (monthly active users [MAUs]), and in the first half of 2020, there were an average of 6 million MAUs of NUGU [18]. NUGU analyzes users’ natural language (voice or text) requests, interprets them, and then provides users with information or related services. For detailed information, see Multimedia Appendix 1.

### Brain Toktok: The Smart Speaker–Based MMT Program

In this study, the smart speaker–based MMT is called “Brain Toktok” or “Brain Opal.” It was commercialized on July 30, 2020. Users of NUGU speakers can subscribe to it [19]. Brain Toktok is based on a multistrategy MMT program developed by Youn and colleagues [7]. Previous offline MMT studies were conducted by trained psychological experts and MMT has already been well-validated [5-7]. The offline MMT program applied several memory strategies based on the metamemory concept. The program consists of 10 weekly sessions. Each session is comprised of three parts, and it takes 90 minutes to complete a session. The goal of the first part of each session is to strengthen meta-knowledge. The second and third parts of each session focus on meta-monitoring, meta-judgment, and memory training via the practice of external and internal strategies. Brain Toktok was developed through a series of analytic stages (content, learner, technology, and environment analysis) and design stages (information, interaction, synchronous, and evaluation design), followed by expert consultation. In this process, the goal of Brain Toktok was to focus on the steady participation of users in training rather than to increase the user’s correctness rate. Finally, 11 cognitive training exercises were developed. Three programs are randomly selected for use among the 11 cognitive training exercises possible per session. The provided training program was selected randomly without a fixed standard, and the content provided for each session was not recorded separately. Users can respond to multiple-choice or open-response questions specific to the programs provided and consist of more than 100 questions per program. For detailed information about the individual programs, see Multimedia Appendix 2.

### Measures

#### Short-Form Geriatric Depression Scale (SGDS)

Many studies have shown a high association between depression and cognitive function, and depression can independently affect cognitive function. The Geriatric Depression Scale (GDS) [20] is one of the most widely used instruments worldwide for screening late-life depression. This scale involves a 30-item easy-to-administer inventory; it has been widely used in both communities and institutions [21,22]. To simplify this screening device for depression, a short-form version was also developed (SGDS), which was extracted from the original GDS. There have been many studies proving that the SGDS is an adequate substitute for the 30-item GDS [23-26]. This scale was standardized for Korean older adults [27]. The SGDS consists of 15 questions, and all questions can be answered with yes or no. A score of 10 or above indicates major depression. Item analysis also confirmed consistency. All 15 items of SGDS were significantly correlated with the full GDS, and its sensitivity (91%) and specificity (82%) were very high [28]. Therefore, the SGDS was used in this study to assess depressed mood in older adults and to exclude patients with major depressive disorder.

#### Subjective Memory Complaints Questionnaire (SMCQ)

The SMCQ is a brief, self-rated questionnaire for assessing subjective memory complaints (SMC), including memory problems in general and daily living. It consists of 14 items reflecting different aspects of SMC, which represents the metacognition of general and specific memories. Four items assess subjective judgment of memory impairment and 10 items assess memory deficit in everyday life. Higher scores indicate a greater perceived cognitive decline. Participants with an SMCQ score of 6 or above were assigned to the SMC group. The SMCQ has been validated and adapted for the Korean population [29]. Consequently, SMCQ is a reliable and important tool for SMC evaluation by measuring subjective cognitive problems. Therefore, we used the SMCQ to measure participants’ SMC at baseline to try to provide homogeneity between the training and control groups. In addition, SMCQ is meaningful in that it can reflect the degree of metamemory because it is measured by the recognition and judgment of its memory function.

#### Mini-Mental State Examination (MMSE)

The MMSE was designed to assess cognitive functioning [30]. The Korean version of the MMSE was developed and validated in older Korean adults by Lee et al [31]. The score ranges from 0 to 30, with higher scores indicating better cognition. The MMSE score reflects functioning in six areas, each scored separately: orientation (10 points), short-term memory registration (3 points), memory recall (3 points), attention and calculation (5 points), language (8 points), and copying a double pentagon (1 point).

#### Verbal Learning Test: Rappel Indicé (RI)

The RI was used to assess verbal learning and memory. The RI was developed by Adam et al [32] and was validated for use with older Korean adults [33]. The RI includes 24 items across six different categories for recall and uses category-cued recall. For example, the fruit category consists of four words: grape, banana, watermelon, and oriental melon. The participants first complete a learning phase in which four items are presented on a slide. Of these, they are asked to name the item included in the semantic category provided by the examiner. For example, for the item “grape,” the examiner would ask, “Which item is in the category of fruit?” Correct answers are then scored, with total scores ranging from 0 to 24. Approximately 20 minutes later, participants are asked to recall the four items from each category in any order. There is no specific time limit, and the examiner proceeds to the next category if the participant is unable to list the remaining items from the category. Correctly recalled answers are scored from 0 to 24. In this study, verbal learning and delayed free recall were assessed using the RI.

#### Working Memory: Digit Span Test

The Digit Span Test is a component of the Elderly Memory Disorder Scale, which has been developed and validated to measure memory and cognitive functions in older Korean adults [34]. The test consists of a forward and backward digit order recall. The examiner calls number digits and asks participants to repeat the list both forward and backward. Scores range from 0 to 14.

#### Executive Function: Fluency Test

The fluency test also comes from the Elderly Memory Disorder Scale [34]. This test evaluates the ability to form and fluently utter words compatible with given criteria. The test consists of three parts. The first section involves listing as many words as possible belonging to a given semantic category in 30 seconds (usually, this is made up of the names of objects). In the second section, the objective is to list as many words as possible belonging to a given phonetic category (ie, words containing a given sound) in 30 seconds. In the third section, the objective is to list as many words associated with a given item as possible in 30 seconds. In this study, the cues were the names of fruits, words containing the Korean letter “Ma,” and words associated with the word “fox.” The score for this test is calculated as the number of correct words listed for each category and item.

### Procedures

This study was conducted from October 2019 to March 2020. Individuals who agreed to participate were assigned into two groups using convenience sampling. The control group did not receive any training; neuropsychological measures were simultaneously taken in the control group and in the training group before and after the training was completed in the latter group. As shown in Figure 1, 6 control group participants were excluded from the study because they did not complete the postevaluation. At the start of the study, participants in the training group were instructed on the use of the smart speaker. Neuropsychological measures (ie, SGDS, SMCQ, MMSE, RI, Digit Span Test, and fluency test) were assessed twice during the study: before the training (pretraining evaluation) and after the training (posttraining evaluation).

The training group received motivation reinforcement education training at the beginning of the smart speaker–based MMT. Group education took place for 1 hour and 30 minutes, and lectures were conducted on the definition, purpose, effectiveness, and necessity of metacognitive training. Afterward, participants had an opportunity to provide feedback. The 8-week training program was designed to include training sessions 3 times a day, 5 days a week (weekdays), for approximately 15 minutes. The patient flexibly set the time without setting a specific time for program participation. During Brain Toktok, the program may be suspended if there are external factors such as personal circumstances of the user or instability of the internet connection. If the program is interrupted due to external factors, it starts again from the beginning when participation resumes. To check the training group’s compliance, the number and duration of times each patient turned on the program and the number of times they completed the session was stored in the server. Participants who did not complete a training session in a given day received an encouraging program message. In the control group, the monitoring system was not implemented as in the experimental group, but the control group was registered in the local welfare center and the situation of the control group was continuously confirmed by the welfare center staff.

All participants provided written consent before study participation. The study was conducted following the Declaration of Helsinki and approved by the ethics review board of the Seoul National University Boramae Medical Center. Participants received a gift card worth US $85 as compensation for their participation in the study.

### Focus Group Interviews

Focus group interviews were conducted with participants in the experimental group to assess their experience with and the effectiveness of the smart speaker–based MMT program. After completing the smart speaker–based MMT course, interviews were conducted with 28 participants who had agreed to the interview during the course. It was composed of eight open-ended questions, and for each question, the subject was asked to freely describe their thoughts about the item. An experienced clinical psychologist conducted the interviews and recorded both their observations and the participants’ responses.

### Statistical Analysis

Statistical analyses were conducted using SPSS software (version 20.0; IBM Corp). Differences between groups in demographics and neuropsychological measures (ie, age, education, gender, and MMSE score) were assessed using independent *t* tests or χ^2^ tests. Repeated-measures analysis of variance (rmANOVA) was used to evaluate differences between the pre-and posttraining neuropsychological scores in the training and control groups. For model I, no adjusted factors were used. For model II, adjusted factors were used for SGDS and SMCQ scores. Because the SGDS and SMCQ scores varied between the groups at baseline, adjusting for the effects of SGDS and SMCQ scores allowed us to see the true effect of MMT on the memory test score. Statistical tests were two-tailed, with *P*<.05 indicating significant results.

## Results

### Participant Characteristics

Figure 1 summarizes the characteristics of the study participants. No difference was found in the drop-out rate between the two groups (MMT group: 9 dropouts, control group: 6 dropouts; χ^2^=0.65; *P*=0.42). All but 6 participants in the MMT group achieved more than 80% compliance with the attendance requirement. The 6 subjects who failed to achieve more than 80% compliance did so because of personal reasons (ie, hospital admission, sickness, preparation for moving, etc). The details of compliance are shown in Multimedia Appendix 2. Table 1 summarizes the demographic and clinical characteristics of the MMT and control groups. There were no differences between the groups regarding gender distribution, age, education, or MMSE scores. There was a difference in the SMCQ and SGDS scores between the groups. Therefore, we adjusted the SMCQ and SGDS scores in regression model II.

**Table 1:** Demographic characteristics of the metamemory training (MMT) and control groups.

Characteristics	Total (N=60)	Groups		Mean difference (95% CI) or χ^2^	*P* value
		MMT (n=29)	Control (n=31)		
Female gender, n (%)	50 (83.3)	24 (82.8)	26 (83.9)	0.01	.91
Age, years (SD)	71.21 (5.72)	70.48 (6.08)	71.94 (5.36)	1.453 (–1.503 to 4.409)	.33
Education, years (SD)	10.60 (3.83)	10.17 (4.17)	11.03 (3.49)	0.860 (–1.121 to 2.841)	.39
SGDS^a^ score, mean (SD)	2.26 (3.37)	3.21 (4.16)	1.32 (2.59)	–1.884 (–3.662 to –0.106)	.04
SMCQ^b^ score, mean (SD)	17.84 (2.64)	18.69 (3.13)	17.00 (2.16)	–1.690 (–3.072 to –0.308)	.02
MMSE^c^ score, mean (SD)	27.90 (1.50)	27.76 (1.66)	28.03 (1.33)	0.274 (–0.501 to 1.049)	.48

^a^SGDS: Short-Form Geriatric Depression Scale.

^b^SMCQ: Subjective Memory Complaints Questionnaire.

^c^MMSE: Mini-Mental State Examination.

### Effects on Improving Cognitive Function

As shown in Table 2, we found significant interactions between group and score change for the tests of delayed free recall, fluency, digit span forward, and digit span backward. Because SMC may affect cognitive performance regardless of memory training, even after an improvement of depressive symptoms, the change in the SGDS score was controlled for by treating it as a covariate. After controlling for the effects of baseline SMCQ and SGDS scores, the interactions remained significant (model II in Table 3). The change in verbal immediate recall score did not interact significantly with the group.

**Figure 2:**
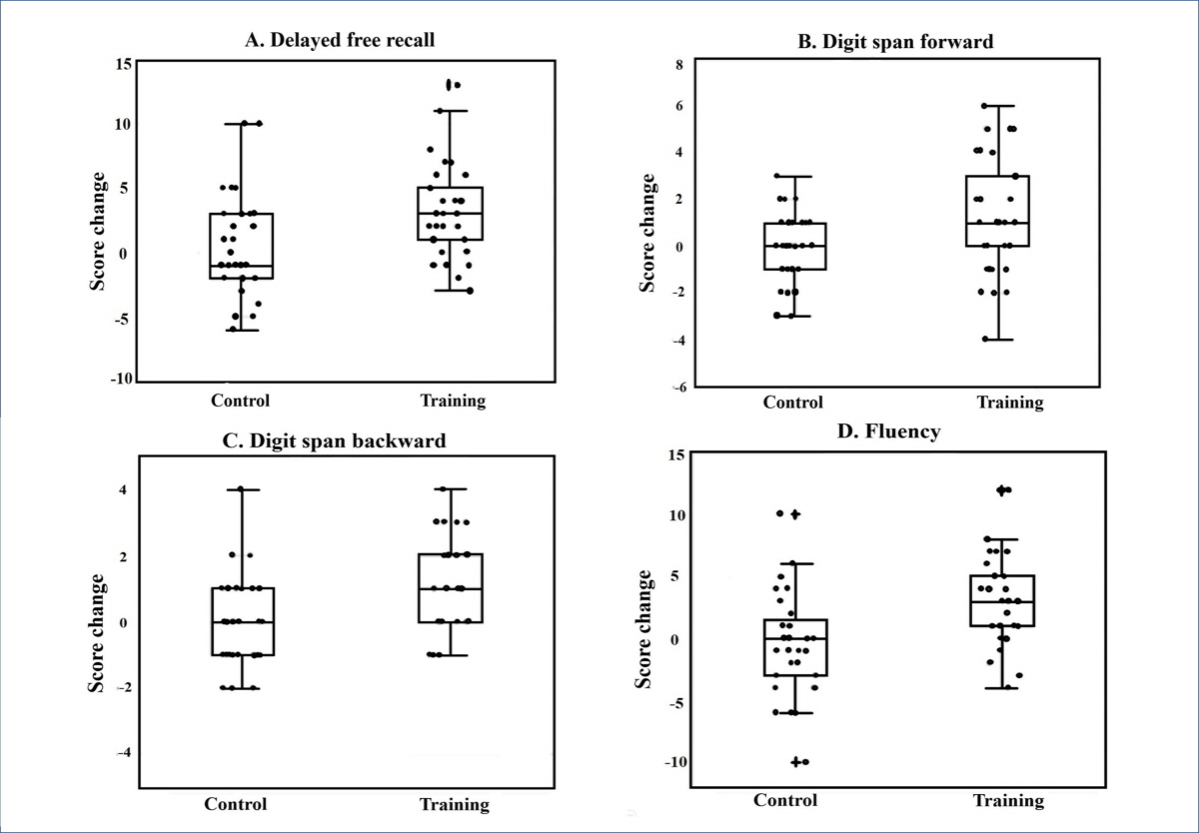
Box and scatter plots of cognitive function changes in metamemory training and control groups during the 8 weeks of the study. (A) Delayed free recall (*P*<.001; *t*=0.50; Cohen *d*=0.84). (B) Digit span forward (*P*=.01; *t*=2.71; Cohen *d*=0.50). (C) Digit span backward (*P*<.001; *t*=4.52; Cohen *d*=0.84). (D) Fluency (*P*<.001; *t*=4.58; Cohen *d*=0.85).

The scores for delayed free recall, fluency, digit span forward, and digit span backward were significantly higher in the group that participated in the MMT program for 8 weeks; there were no significant cognitive changes in the control group after 8 weeks (Tables 2 and 3, and Figure 2).

**Table 2:** Changes in cognitive function in the metamemory training (MMT) and control groups.

Cognitive function (measures)		MMT group (n=29)			Paired *t* test			Control group (n=31)				Paired *t* test		
		Pre, mean (SD)	Post, mean (SD)	MD^a^ (95% CI)		MMT group (*d*^b^ 1)	Control group (*P* value 1)		Pre, mean (SD)	Post, mean (SD)	MD (95% CI)		MMT group (*d* 2)	Control group (*P* value 2)
**Verbal memory**														
	Learning	22.03 (4.49)	22.00 (2.45)	0.03 (–1.87 to 1.94)		.01	.97		23.23 (1.43)	23.45 (1.18)	–0.23 (–0.89 to 0.44)		.12	.52
	Delayed cued recall	12.21 (4.47)	15.34 (3.71)	–3.14 (–5.3 to –0.98)		.84	<.001		13.00 (3.92)	13.35 (3.73)	–0.36 (–2.3 to 1.59)		.09	.62
**Attention/executive functions**														
	Digit span forward	5.97 (1.64)	7.24 (2.26)	–1.28 (–2.32 to –0.24)		.50	.01		6.65 (1.58)	6.68 (1.11)	–0.03 (–0.73 to 0.66)		.02	.90
	Digit span backward	3.41 (1.38)	4.55 (1.38)	–1.14 (–1.86 to –0.41)		.84	<.001		3.70 (1.32)	3.84 (1.34)	–0.1 (–0.77 to 0.58)		.08	.69
**Language**														
	Fluency	15.76 (3.90)	18.86 (4.35)	–3.10 (–5.28 to –0.93)		.85	<.001		18.03 (4.50)	17.55 (5.00)	0.48 (–1.93 to 2.90)		.12	.51

^a^MD: mean difference.

^b^Cohen *d*.

**Table 3:** Interaction between cognitive changes and study groups.

Measures of cognitive function		Main effect of cognitive change/interaction between cognitive change and study group					
		Model I^a^			Model II^b^		
		*F*	η^2^	*P* value	*F*	η^2^	*P* value
**Verbal memory**							
	Learning	0.03/0.06	0.001/0.001	.86/.81	0.13/0.19	0.002/0.003	.72/.67
	Delayed free recall	12.38/7.86	0.18/0.12	.001/.007	0.22/8.64	0.004/0.13	.64/.005
**Attention/ executive functions**							
	Digit span forward	6.08/5.49	0.10/0.09	.02/.02	1.75/4.15	0.03/0.07	.19/.046
	Digit span backward	12.72/9.04	0.18/0.14	.001/.004	0.42/6.19	0.007/0.10	.52/.02
**Language**							
	Fluency	6.90/12.94	0.11/0.18	.01/.001	0.75/9.24	0.01/0.14	.39/.004

^a^Model I: no adjusted factors.

^b^Model II: adjusted for Short-Form Geriatric Depression Scale and Subjective Memory Complaints Questionnaire scores.

### Focus Group Interviews

Users of smart speakers reported that they were easier to use and less burdensome than other information and communications technology tools such as computers and mobile phones. For example, one user reported, “The smart speaker was convenient because I did not need to press the keyboard or the button, and usually I didn’t use the cell phone very well. Rather, I used it more frequently than the cell phone.”

Although the smart speaker is a machine, users reported that communicating with it was like a human-to-human conversation: “Television is what you see, and you can't talk but the smart speaker is able to communicate.” Some users preferred to communicate with the smart speaker rather than real people, and they enjoyed it: “If I get it wrong repeatedly, people get easily tired. The AI speaker keeps listening and encouraging;” “Smart speakers feel like comfortable friends, sometimes like a family;” and “I don't like to communicate to people very much, but I enjoyed communicating to the smart speaker when I needed it.”

## Discussion

### Principal Results

According to the study results, our smart speaker–based MMT program improved memory ability, executive ability, and working memory. After adjusting for SMCQ and SGDS scores, the effects were still significant. These results indicate that cognitive improvements may be achieved through the effects of pure cognitive training. Furthermore, smart speaker–based MMT, which can easily be carried out without trained experts, fostered high compliance. Hence, it offers a good cognitive training tool without the typical limitations imposed by location or time constraints. To the best of our knowledge, this is the first report on a cognitive training program using a smart speaker. We hope that this study offers a useful guideline for future research on cognitive training programs based on smart speakers.

### Comparison with Prior Research

Previous studies on the effectiveness of smart speaker–based MMT reported that healthy older adults achieved improvements in memory ability, verbal fluency performance, and working memory [7]. The results of this study are similar to those of previous studies. Users of smart speaker–based MMT may still experience the same cognitive improvements as people who undertake in-person courses conducted by experts.

### Effects on Delayed Free Recall and Verbal Memory

In this study, the scores for delayed free recall and verbal memory were significantly higher in this healthy older adult population after the training. Decreased delayed recall scores are considered the best predictor of progression from a dementia-free period to Alzheimer disease dementia (ADD) [35]. A previous study reported that expert MMT was associated with significantly improved cognition in subjects with mild cognitive impairment (MCI) [6]. These results suggested that smart speaker–based MMT may slow not only cognitive decline in healthy individuals but the progression of MCI to ADD.

### Effects on Verbal Fluency

Among the improvements in executive functions, the improvement in verbal fluency was significant. Previous studies have reported that verbal fluency was significantly associated with social activity [36]. According to the US Census Bureau, in 2010, 30% of people aged 65 years and older lived alone at the time of the census. As people get older, their likelihood of living alone increases [37]. Due to the social conditions associated with aging, older people are more likely to be vulnerable to verbal fluency problems. Smart speaker–based MMT can effectively support the vulnerable cognitive functioning of older adults who live alone. Also, older people who live alone and repeat relatively simple life patterns have a higher risk of depression and poor cognitive abilities compared with older people who do not-live alone. Smart speaker–based MMT is conducted through conversations with users, which can serve as a new stimulus for the elderly user who lives alone [38]. According to our focus group interviews, smart speaker users reported that communicating with it was like conversing with another person, so our smart speaker–based MMT may help those who live alone.

### Effects on Digit Span Forward and Backward

Digit span forward and backward tests are the most commonly used tests in clinical neuropsychology to assess working memory capacity [39]. Working memory is a key focus of memory improvement strategies. It can be trained through smart speaker–based MMT and then employed to increase cognitive reserve to prevent dementia. Furthermore, working memory is one of the core mechanisms involved in higher-order cognitive abilities, such as problem-solving, fluid intelligence, and reading comprehension [40,41]. It is also one of the cognitive processes that suffers a clear and linear decline with aging [42,43]. In this study, digit span forward and backward tests were significantly improved in the group that participated in 8 weeks of training. Smart speaker–based MMT may help maintain or improve higher-order cognitive abilities by enhancing working memory capacity.

### Limitations

There are some limitations to this study, the first being the small sample size. However, this is the first time a cognitive training program has been developed using the smart speaker, so this paper provides meaningful guidelines for future studies. Second, this study did not adopt an active control design, and the control group took no action. Further studies should use an active control design. Third, the SMCQ and SGDS scores were not homogenous between the groups. To remove the effects of SMCQ and SGDS scores, the SMCQ and SGDS values were adjusted during the analysis. We found that the efficacy of the MMT program using the smart speaker was not affected by the SMCQ and SGDS scores. Therefore, the results of this study are reliable.

Fourth, neuropsychological tests performed before and after the experiment are not a direct measure of metamemory function. Still, we used neuropsychological tests to assess the functioning of general memory areas. Specifically, the test of cued recall is the most effective test of memory function status. We also employed the cued recall test to discover the effectiveness of smart speaker–based-MMT. Although a direct measure of metamemory function was not used, the purpose of this study was to examine the enhancement of memory function through MMT. We consider the study methodology to be suitable for its purpose. Finally, we assigned participants to the two study groups by convenience sampling. We are aware of one other recent study that has addressed this issue [44]. Although cross-sectional in design, the previous study found few significant differences when contrasting the demographic characteristics and psychological performance of older adults in a random sample instead of two convenience samples. Specifically, the previous study observed no differences in word list recall results between the random and convenience samples recruited for a study of memory and aging. In further studies, to increase the power and proof of the effectiveness of smart speaker–based MMT, a large sample size and a more elaborate research design such as a randomized controlled study with an active control design should be implemented.

### Conclusion

Smart speaker–based MMT, without location requirements and time constraints, may address older adult memory problems and possibly improve their quality of life by helping them cope with the cognitive issues associated with aging. Our smart speaker–based MMT may also be useful in delaying the onset of dementia.

Our study’s strength is that it is the first report on a cognitive training program using a smart speaker. In the future, the use of smart speakers will be more commercialized, expanding with the paradigm shift to “social distancing.” The problem of memory deterioration due to the increase in the elderly population will become more critical. In the future, the combination of a smart speaker and the memory training program will be of great help to older adults. We hope that this study offers a useful guideline for future studies on cognitive training programs based on smart speakers.

## Appendix

Multimedia Appendix 1 NUGU: Smart speaker platform.

Multimedia Appendix 2 Supplementary table.

## Abbreviations

ADD: Alzheimer disease dementia
AI: artificial intelligence
GDS: Geriatric Depression Scale
MAU: monthly active user
MCI: mild cognitive impairment
MMSE: Mini-Mental State Examination
MMT: metamemory training
RI: Rappel Indicé
rmANOVA: repeated-measures analysis of variance
SGDS: Short-Form Geriatric Depression Scale
SLDS: spoken language dialogue system
SMC: subjective memory complaints
SMCQ: Subjective Memory Complaints Questionnaire

## References

[1] Flavell JH (1979). Metacognition and cognitive monitoring: A new area of cognitive-developmental inquiry. American Psychologist.

[2] Hertzog C, Dixon RA, Hultsch DF (1990). Relationships between metamemory, memory predictions, and memory task performance in adults. Psychol Aging.

[3] Gilleen J, David A, Greenwood K (2016). Self-reflection and set-shifting mediate awareness in cognitively preserved schizophrenia patients. Cogn Neuropsychiatry.

[4] McDougall Graham J, Kang J (2003). Memory Self-Efficacy and Memory Performance in Older Males. Int J Mens Health.

[5] Youn J, Ryu S, Lee J, Park S, Cho S, Kwon H, Yang J, Lee J, Lee J, Kim S, Livingston G, Yoon DH (2019). Brain structural changes after multi-strategic metamemory training in older adults with subjective memory complaints: A randomized controlled trial. Brain Behav.

[6] Youn J, Park S, Lee J, Cho S, Kim J, Ryu S (2020). Cognitive Improvement in Older Adults with Mild Cognitive Impairment: Evidence from a Multi-Strategic Metamemory Training. J Clin Med.

[7] Youn J, Lee J, Kim S, Ryu S (2011). Multistrategic memory training with the metamemory concept in healthy older adults. Psychiatry Investig.

[8] Sarah Perez COVID-19 quarantine boosts smart speaker usage among U.S. adults, particularly younger users. techcrunch.

[9] Kandalaft MR, Didehbani N, Krawczyk DC, Allen TT, Chapman SB (2013). Virtual reality social cognition training for young adults with high-functioning autism. J Autism Dev Disord.

[10] Yang YJD, Allen T, Abdullahi SM, Pelphrey KA, Volkmar FR, Chapman SB (2018). Neural mechanisms of behavioral change in young adults with high-functioning autism receiving virtual reality social cognition training: A pilot study. Autism Res.

[11] Man DWK, Poon WS, Lam C (2013). The effectiveness of artificial intelligent 3-D virtual reality vocational problem-solving training in enhancing employment opportunities for people with traumatic brain injury. Brain Inj.

[12] Vogan AA, Alnajjar F, Gochoo M, Khalid S (2020). Robots, AI, and Cognitive Training in an Era of Mass Age-Related Cognitive Decline: A Systematic Review. IEEE Access.

[13] Bertrand G, Nothdurft F, Minker W (2012). What do you want to do next? Providing the user with more freedom in adaptive spoken dialogue systems.

[14] Stolcke A, Droppo J (2017). Comparing human and machine errors in conversational speech transcription.

[15] Oord AVD, Dieleman S, Zen H, Simonyan K, Vinyals O, Graves A, Kalchbrenner N, Senior A, Kavukcuoglu K (2016). WaveNet: A Generative Model for Raw Audio Based on PixelCNN Architecture. arXiv 2016.

[16] Schnelle D, Lyardet F (2006). Voice user interface design patterns.

[17] SK Telecom Group SKT NUGU. SK telecom Co.

[18] Hayoung Kim (2019). The 3rd anniversary of the launch of SKT “NUGU”... “6.7 million users more than once a month.”. Digital Daily.

[19] Huegang Shin SKT, Lanched “NUGU opal”, AI service for older adults. Newdaily.

[20] Yesavage JA, Brink TL, Rose TL, Lum O, Huang V, Adey M, Leirer VO (1982). Development and validation of a geriatric depression screening scale: a preliminary report. J Psychiatr Res.

[21] Hyer L, Blount J (1984). Concurrent and discriminant validities of the geriatric depression scale with older psychiatric inpatients. Psychol Rep.

[22] Zgourides G, Spofford M, Doppelt L (1989). The Geriatric Depression Scale: discriminant validity and elderly day-treatment clients. Psychol Rep.

[23] Sherry A Greenberg (2019). The geriatric depression scale (GDS) validation of a geriatric depression screening scale: A preliminary report. Best Pract Nurs Care to Older Adults.

[24] Lesher EL, Berryhill JS (1994). Validation of the geriatric depression scale-short form among inpatients. J. Clin. Psychol.

[25] Fountoulakis KN, Tsolaki M, Iacovides A, Yesavage J, O'Hara R, Kazis A, Ierodiakonou C (1999). The validation of the short form of the Geriatric Depression Scale (GDS) in Greece. Aging (Milano).

[26] Burke WJ, Roccaforte WH, Wengel SP (1991). The short form of the Geriatric Depression Scale: a comparison with the 30-item form. J Geriatr Psychiatry Neurol.

[27] Bae JN, Cho MJ (2004). Development of the Korean version of the Geriatric Depression Scale and its short form among elderly psychiatric patients. J Psychosom Res.

[28] Cho M, Bae J, Suh G, Hahm B, Kim J, Lee D, Kang M (1999). Validation of Geriatric Depression Scale, Korean Version(GDS) in the Assessment of DSM-III-R Major Depression. J Korean Neuropsychiatr Assoc.

[29] Youn JC, Kim KW, Lee DY, Jhoo JH, Lee SB, Park JH, Choi EA, Choe JY, Jeong JW, Choo IH, Woo JI (2009). Development of the Subjective Memory Complaints Questionnaire. Dement Geriatr Cogn Disord.

[30] Folstein MF, Folstein SE, McHugh PR (1975). "Mini-mental state". A practical method for grading the cognitive state of patients for the clinician. J Psychiatr Res.

[31] Lee DY, Lee KU, Lee JH, Jhoo JH, Kim KW, Youn JC, Kim SY, Woo SI, Woo JI (2002). A Normative Study of the Mini-Mental State Examination in the Korean Elderly. J Korean Neuropsychiatr Assoc.

[32] Adam S, Van der Linden M, Ivanoiu A, Juillerat A, Bechet S, Salmon E (2007). Optimization of encoding specificity for the diagnosis of early AD: the RI-48 task. J Clin Exp Neuropsychol.

[33] Park S, Kim I, Park HG, Shin SA, Cho Y, Youn J, Kim YK, Lee J (2016). Development and Validation of the Rappel Indicé-24: Behavioral and Brain Morphological Evidence. J Geriatr Psychiatry Neurol.

[34] Choi JY (2007). Elderly memory disorder scale.

[35] Elias MF, Beiser A, Wolf PA, Au R, White RF, D'Agostino RB (2000). The preclinical phase of alzheimer disease: A 22-year prospective study of the Framingham Cohort. Arch Neurol.

[36] Kelly ME, Duff H, Kelly S, McHugh Power JE, Brennan S, Lawlor BA, Loughrey DG (2017). The impact of social activities, social networks, social support and social relationships on the cognitive functioning of healthy older adults: a systematic review. Syst Rev.

[37] Census Bureau United States United states Census Bureau in 2010 Internet. United States Census Bureau.

[38] Jung Y, Kim J (2004). [Comparison of cognitive levels, nutritional status, depression in the elderly according to living situations]. Taehan Kanho Hakhoe Chi.

[39] Hilbert S, Nakagawa TT, Puci P, Zech A, Bühner M (2015). The Digit Span Backwards Task. European Journal of Psychological Assessment.

[40] Borella E, Ghisletta P, de Ribaupierre A (2011). Age differences in text processing: the role of working memory, inhibition, and processing speed. J Gerontol B Psychol Sci Soc Sci.

[41] Graf H-P, Ohta N (2002). Lifespan Development of Human Memory.

[42] Borella E, Carretti B, De Beni R (2008). Working memory and inhibition across the adult life-span. Acta Psychol (Amst).

[43] Mammarella IC, Borella E, Pastore M, Pazzaglia F (2013). The structure of visuospatial memory in adulthood. Learning and Individual Differences.

[44] Hultsch DF, MacDonald SW, Hunter MA, Maitland SB, Dixon RA (2016). Sampling and generalisability in developmental research: Comparison of random and convenience samples of older adults. International Journal of Behavioral Development.

