# Supplementary material for: Efficacy of Smart Speaker–Based Metamemory Training in Older Adults: Case-Control Cohort Study
Source: J Med Internet Res. 2021 Feb 16;23(2):e20177. doi: 10.2196/20177 (PMC7925152; doi:10.2196/20177)

**Multimedia Appendix 1.** NUGU: Smart speaker platform*.*

This study was conducted using a NUGU speaker (see multimedia appendix Figure 1) widely used in Korea. NUGU is a smart speaker platform developed by Korea's leading mobile communication company - SK Telecom company. NUGU analyzes users’ natural language (voice or text) requests, interprets them, and then provides information or related services to users. The NUGU platform is used for various points of contact, such as the NUGU speaker, the Internet protocol television and coding robot Albert, and others.

The NUGU speaker provides functionality from the NUGU open platform, such as automatic speech recognition, natural language understanding/generation, text–to–speech transformation, and dialog management (see multimedia appendix Figure 2). The NUGU speaker contains Android-platform-frameworks–base library, which can be downloaded from https://android.googlesource.com/platform/frameworks/base. This library includes the fol-lowing license and notice below: Copyright (C) 2008 The Android Open Source Project Licensed under the Apache License, Version 2.0 (the "License").

User utterances are managed by the NUGU platform as follows: i) NUGU analyzes the user's intention through voice recognition, natural language understanding; ii) the dialog manager performs the appropriate action in response to the identified request and generates the response to be delivered to the user; and iii) the generated response is transmitted to the user as a synthesized sound, which is provided by the voice synthesis module. ‘Play Builder’ is an integrated development environment that provides the necessary elements for developing user utterance processing services. We implemented the smart speaker–based metamemory training program in Play Builder, using the ‘Private Play’ option (i.e., used only by s specific user/device). The detail machine learning algorithms that were used in this technology was descripted in previous study [1].

Reference

1. Myungsoon P. Study on the factors affecting the continuous use intention of intelligent personal assiatant (Master’s thesis). Seoul: Yonsei University; 2020.

Multimedia appendix figure 1. NUGU speaker


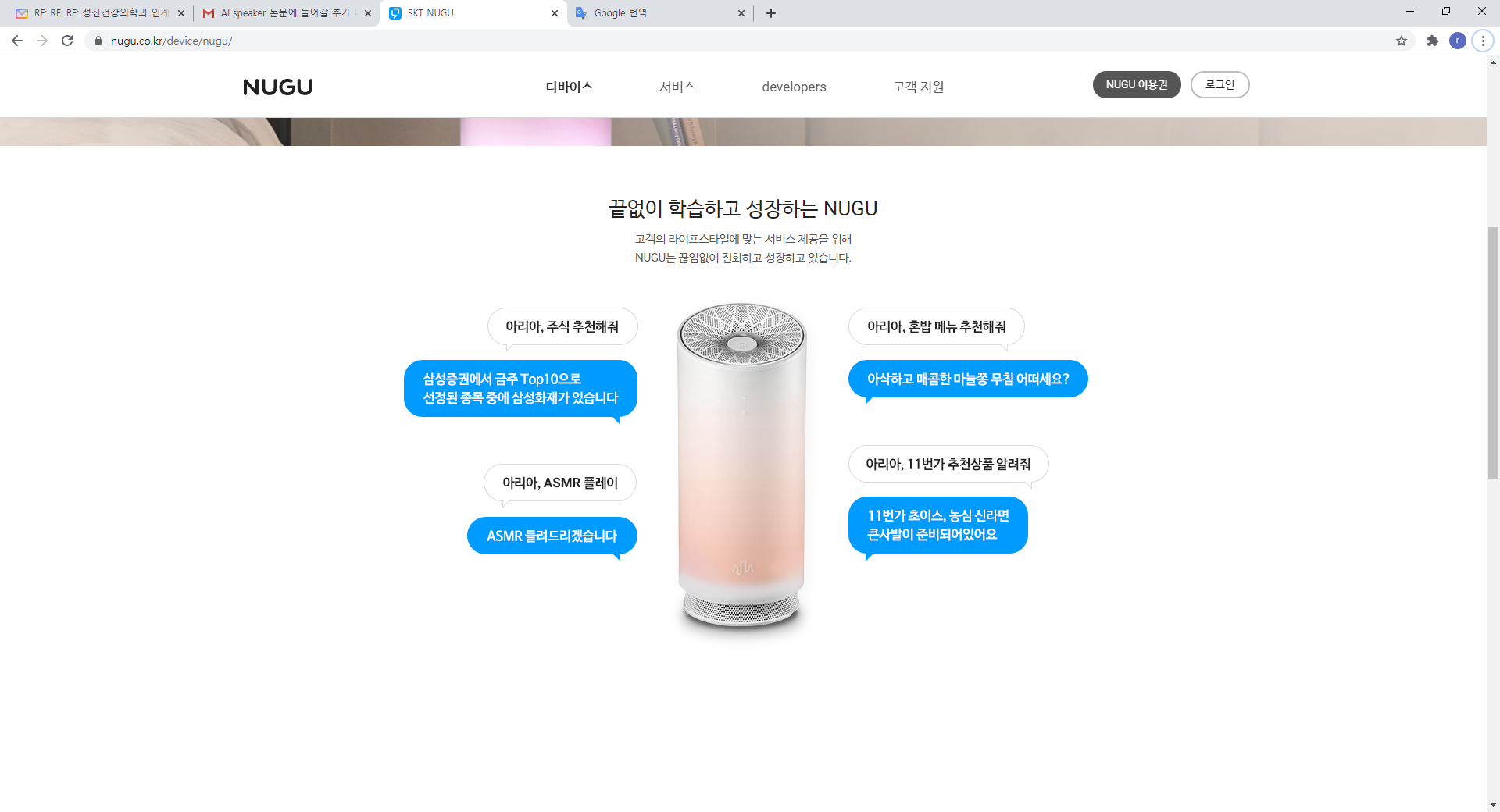

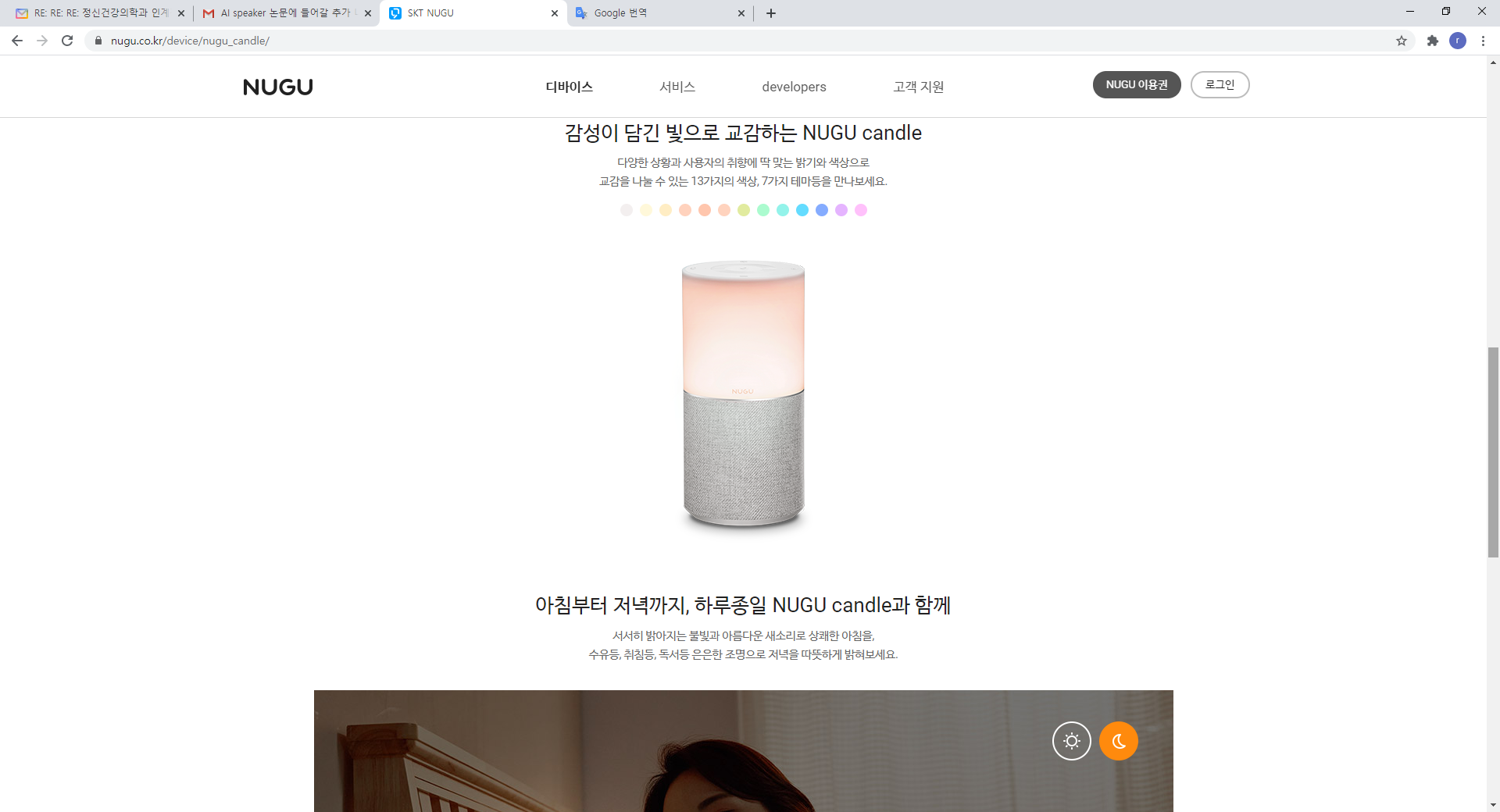

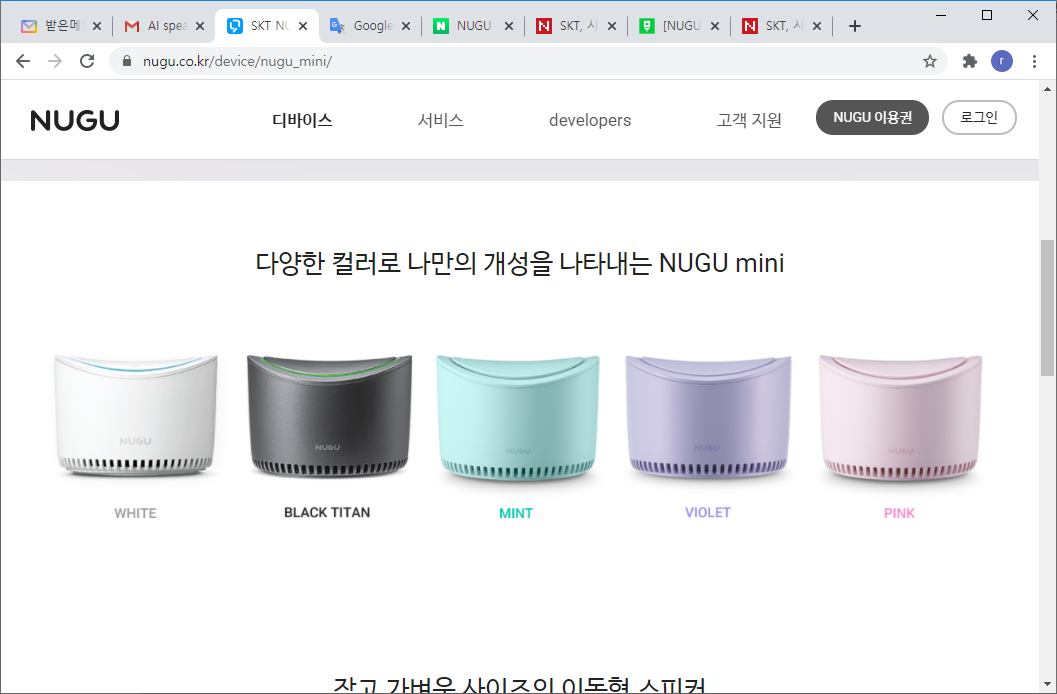

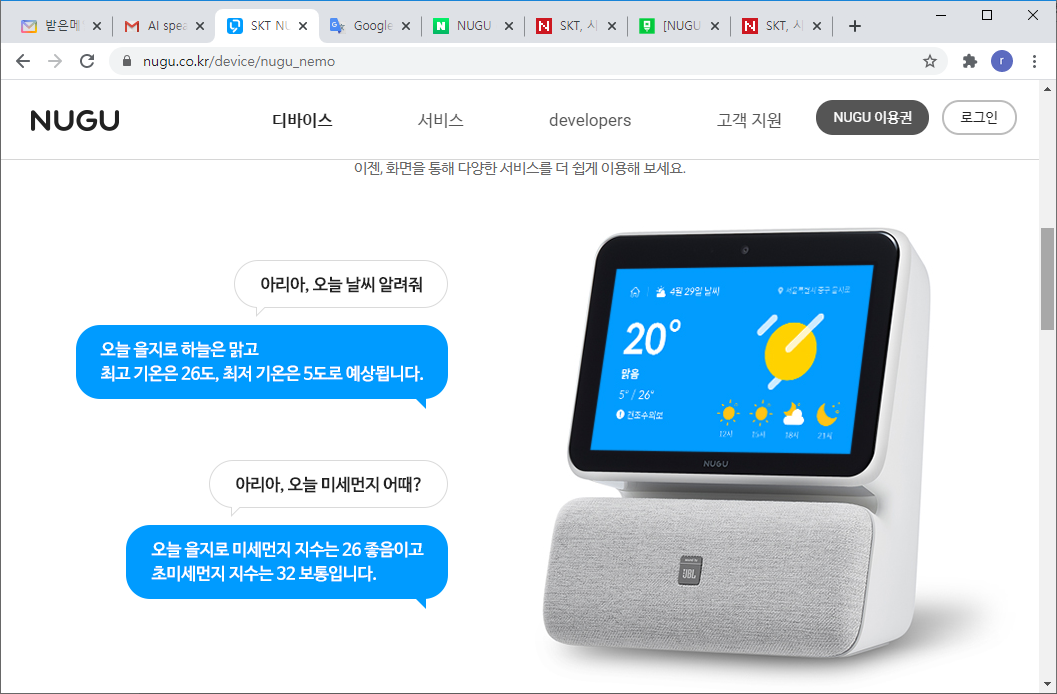


1. NUGU100 B) NUGU 200 C) NUGU 300

Multimedia appendix figure 2. Diagram of NUGU open platform


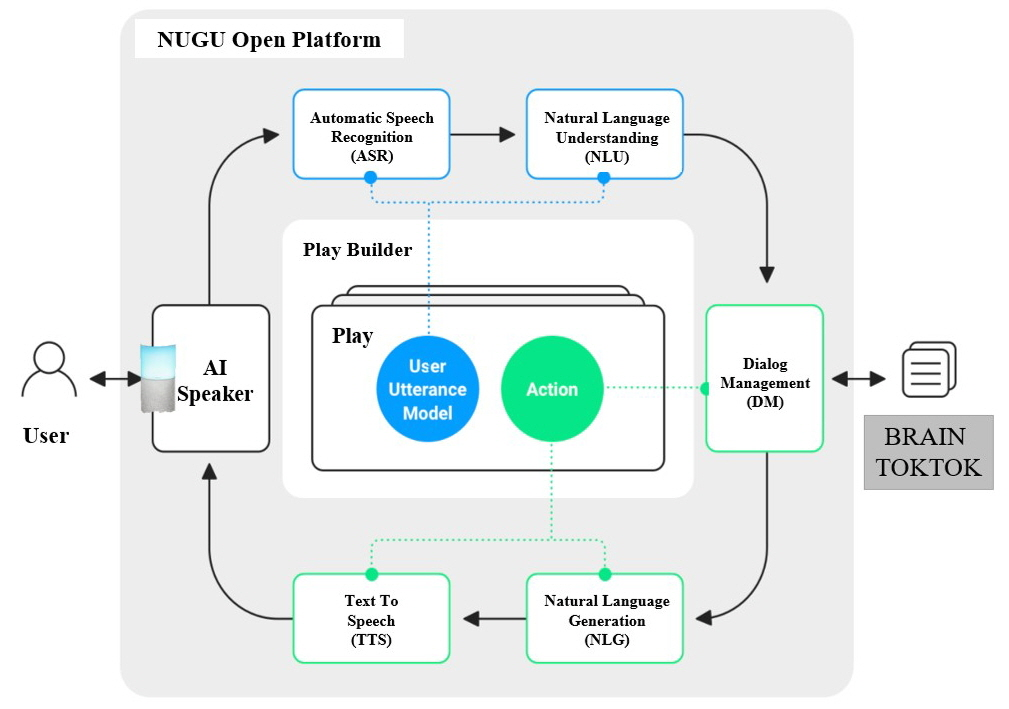

Supplement: Multimedia Appendix 1 [file jmir_v23i2e20177_app1.docx]
