# Supplementary material for: Efficacy of Smart Speaker–Based Metamemory Training in Older Adults: Case-Control Cohort Study
Source: J Med Internet Res. 2021 Feb 16;23(2):e20177. doi: 10.2196/20177 (PMC7925152; doi:10.2196/20177)
Supplement: Multimedia Appendix 2 [file jmir_v23i2e20177_app2.docx]

Multimedia appendix 2

Supplementary table 1. The contents of a smart speaker–based metamemory training program.

| **No.** | **Content** | **Program composition** | **Example** | | **Goal Function** | **Number**  **of questions** |
| --- | --- | --- | --- | --- | --- | --- |
|  |  |  | **question** | **Response (s)** |  |  |
| 1(s) | Presents a topic that the user is familiar with and asks for words related to the topic | First, the smart speaker suggests three words related to the topic, and the user is required to answer three other words related to the topic. The total number of topic is seventeen. In each program, five of seventeen questions are chosen randomly | “Let's remember the familiar words.  I'll tell you three flower names. Sun flower, forsythia, and rose. Now, tell me three flower names.” | “Tulip, cosmos, lily” | *Fluency* | *more than 2000* |
| 2(s) | Quizzes with  3 clues | The total number of questions prepared in each session is five. The total number of question is one hundred. Questions are randomly selected | “From now on, I will give a three-clue quiz. Guess what it is. It's a fruit, it's yellow, and it reminds me of monkeys.  What is this?” | “banana” | *Inference* | *100* |
| 3(M) | Tells a story. Then, gives simple quizzes to recall the story | The total number of stories presented is two in each session. Each story gives three simple quizzes. The total number of stories is forty. Stories are randomly selected. | “I will tell you a story. After finishing the story, I will give you simple quizzes, so listen carefully”   - Tells a story-   “Please tell me ‘yes’ or ‘no’. Was there something related to snakes in the story. | - | *Attention*  *& Memory* | *100* |
| 4(M) | Increases user`s thinking flexibility through nonsense quizzes | The total number of nonsense quizzes presented is three in each session. The total number of Nonsense quizzes are one hundred. Nonsense quizzes is randomly selected. | Let`s answer a nonsense quiz. What is “the fastest bird in the world” called? | - | *Inference* | *100* |
| 5(M) | Asks users to finding the common characteristics of the four given words | The total number of questions presented is five in each session. The total number of questions is one hundred. Questions are randomly selected. | “I'll tell you four words from now on. Find something in common. ‘Warm’, ‘Flower’, ‘green’, ‘sprout’ | - | *Categorization* | *100* |
| 6(s) | Gives a familiar phrase, and then requests the number of specific letters that were repeated. | The total number of questions presented is five in each session. The total number of questions is one hundred. Questions are randomly selected. | “How many times does the word ‘Jang’ repeated in ‘Gan-Jang, Gong-Jang, Gong-Jang-Jang’? | “four” | *Working Memory* | *100* |
| 7(M) | Ask to find the appropriate word that can come after a given word | The total number of questions presented is five in each session. The total number of questions is one hundred. Questions are randomly selected. | “Tell me an appropriate word that can come after the word ‘Merry~’” | *-* | *Confederation* | *100* |
| 8(M) | Asks to find the one word that is different from the other three words among the four presented words. | The total number of questions presented is five in each session. The total number of questions is one hundred. Questions are randomly selected. | “I will give four words, ‘Apple’, ‘Carrot’, ‘Strawberry’, ‘Orange’. Among these four words that are presented, there is one word that is different from the other three. What is it?” | *-* | *Categorization* | *100* |
| 9(M) | Asks to complete one appropriate sentence using the suggested phrase. | The total number of questions presented is five in each session. The total number of questions is one hundred. Questions are randomly selected. | “Please, complete the sentence that starts with the given phrase. ‘The clear sky makes~’” | *-* | *Confederation* | *100* |
| 10(M) | Asks to provide a word associated with a given word. | The total number of questions presented is five in each session. The total number of questions are one hundred. Questions are randomly selected. | “Tell me another word that comes to mind when you listen to the following word. ‘Marriage’” | *-* | *Confederation* | *100* |
| 11(s) | Asks to complete a simple arithmetic calculation | The total number of questions presented is five in each session. The total number of questions is one hundred. Questions are randomly selected. | “What is 5 plus 7?” | “12” | *Attention* | *100* |

(s) : subjective response

(M) : multiple-choice response

Supplementary table 2. Compliance of training group (15 per week is 100%).

| ID | Total | | 1 week | | 2 week | | 3 week | | 4 week | | 5 week | | 6 week | | 7 week | | 8 week | |
| --- | --- | --- | --- | --- | --- | --- | --- | --- | --- | --- | --- | --- | --- | --- | --- | --- | --- | --- |
|  | %^a^ | No.^b^ | % | No. | % | No. | % | No. | % | No. | % | No. | % | No. | % | No. | % | No. |
| 24 | 107% | 128 | 340% | 51 | 160% | 24 | 67% | 10 | 0% | 0 | 47% | 7 | 87% | 13 | 0% | 0 | 153% | 23 |
| 25 | 273% | 328 | 153% | 23 | 313% | 47 | 373% | 56 | 247% | 37 | 120% | 18 | 260% | 39 | 380% | 57 | 340% | 51 |
| 26 | 93% | 112 | 153% | 23 | 140% | 21 | 147% | 22 | 80% | 12 | 47% | 7 | 140% | 21 | 20% | 3 | 20% | 3 |
| 27 | 133% | 159 | 80% | 12 | 0% | - | 20% | 3 | 107% | 16 | 207% | 31 | 27% | 4 | 187% | 28 | 433% | 65 |
| 28 | 148% | 177 | 127% | 19 | 333% | 50 | 207% | 31 | 167% | 25 | 67% | 10 | 80% | 12 | 140% | 21 | 60% | 9 |
| 29 | 176% | 211 | 180% | 27 | 80% | 12 | 200% | 30 | 200% | 30 | 60% | 9 | 193% | 29 | 240% | 36 | 253% | 38 |
| 30 | 188% | 226 | 113% | 17 | 187% | 28 | 207% | 31 | 187% | 28 | 60% | 9 | 180% | 27 | 233% | 35 | 340% | 51 |
| 35 | 143% | 172 | 0% | 0 | 400% | 60 | 307% | 46 | 140% | 21 | 100% | 15 | 93% | 14 | 7% | 1 | 100% | 15 |
| 31 | 475% | 570 | 440% | 66 | 747% | 112 | 720% | 108 | 400% | 60 | 200% | 30 | 420% | 63 | 433% | 65 | 440% | 66 |
| 32 | 269% | 323 | 300% | 45 | 367% | 55 | 360% | 54 | 240% | 36 | 193% | 29 | 260% | 39 | 240% | 36 | 193% | 29 |
| 33 | 234% | 281 | 267% | 40 | 300% | 45 | 320% | 48 | 293% | 44 | 140% | 21 | 293% | 44 | 140% | 21 | 120% | 18 |
| 34 | 107% | 128 | 20% | 3 | 140% | 21 | 60% | 9 | 47% | 7 | 80% | 12 | 80% | 12 | 87% | 13 | 340% | 51 |
| 36 | 146% | 175 | 27% | 4 | 240% | 36 | 313% | 47 | 267% | 40 | 120% | 18 | 80% | 12 | 40% | 6 | 80% | 12 |
| 37 | 302% | 362 | 200% | 30 | 307% | 46 | 100% | 15 | 100% | 15 | 7% | 1 | 393% | 59 | 727% | 109 | 580% | 87 |
| 38 | 164% | 197 | 147% | 22 | 427% | 64 | 280% | 42 | 40% | 6 | 180% | 27 | 40% | 6 | 133% | 20 | 67% | 10 |
| 39 | 134% | 161 | 260% | 39 | 233% | 35 | 127% | 19 | 47% | 7 | 107% | 16 | 107% | 16 | 147% | 22 | 47% | 7 |
| 40 | 296% | 355 | 167% | 25 | 367% | 55 | 333% | 50 | 273% | 41 | 547% | 82 | 373% | 56 | 307% | 46 | 0% | 0 |
| 12 | 345% | 414 | 180% | 27 | 307% | 46 | 287% | 43 | 313% | 47 | 833% | 125 | 413% | 62 | 107% | 16 | 320% | 48 |
| 2 | 231% | 277 | 87% | 13 | 193% | 29 | 253% | 38 | 220% | 33 | 573% | 86 | 180% | 27 | 180% | 27 | 160% | 24 |
| 4 | 158% | 190 | 187% | 28 | 80% | 12 | 107% | 16 | 87% | 13 | 307% | 46 | 140% | 21 | 180% | 27 | 180% | 27 |
| 10 | 3544% | 4253 | 2653% | 398 | 3413% | 512 | 3787% | 568 | 1153% | 173 | 6333% | 950 | 3400% | 510 | 3867% | 580 | 3747% | 562 |
| 11 | 298% | 358 | 233% | 35 | 360% | 54 | 273% | 41 | 280% | 42 | 680% | 102 | 153% | 23 | 153% | 23 | 253% | 38 |
| 13 | 399% | 479 | 413% | 62 | 440% | 66 | 373% | 56 | 240% | 36 | 680% | 102 | 360% | 54 | 400% | 60 | 287% | 43 |
| 15 | 128% | 153 | 53% | 8 | 160% | 24 | 120% | 18 | 113% | 17 | 313% | 47 | 80% | 12 | 60% | 9 | 120% | 18 |
| 17 | 103% | 123 | 93% | 14 | 60% | 9 | 100% | 15 | 200% | 30 | 120% | 18 | 120% | 18 | 107% | 16 | 20% | 3 |
| 18 | 220% | 264 | 0% | 0 | 0% | 0 | 180% | 27 | 460% | 69 | 380% | 57 | 320% | 48 | 320% | 48 | 100% | 15 |
| 19 | 262% | 314 | 200% | 30 | 260% | 39 | 167% | 25 | 627% | 94 | 153% | 23 | 240% | 36 | 387% | 58 | 60% | 9 |
| 20 | 127% | 152 | 0% | 0 | 113% | 17 | 73% | 11 | 253% | 38 | 180% | 27 | 107% | 16 | 227% | 34 | 60% | 9 |
| 22 | 82% | 98 | 40% | 6 | 27% | 4 | 107% | 16 | 107% | 16 | 100% | 15 | 107% | 16 | 87% | 13 | 80% | 12 |
| 23 | 48% | 57 | 67% | 10 | 113% | 17 | 120% | 18 | 40% | 6 | 40% | 6 | 0% | 0 | 0% | 0 | 0% | 0 |
| 3 | 48% | 57 | 20% | 3 | 40% | 6 | 20% | 3 | 60% | 9 | 100% | 15 | 33% | 5 | 53% | 8 | 53% | 8 |
| 5 | 63% | 75 | 133% | 20 | 87% | 13 | 0% | 0 | 7% | 1 | 27% | 4 | 47% | 7 | 60% | 9 | 140% | 21 |
| 8 | 3% | 3 | 0% | 0 | 20% | 3 | 0% | 0 | 0% | 0 | 0% | 0 | 0% | 0 | 0% | 0 | 0% | 0 |
| 9 | 28% | 34 | 20% | 3 | 40% | 6 | 53% | 8 | 20% | 3 | 93% | 14 | 0% | 0 | 0% | 0 | 0% | 0 |
| 14 | 4% | 5 | 13% | 2 | 20% | 3 | 0% | 0 | 0% | 0 | 0% | 0 | 0% | 0 | 0% | 0 | 0% | 0 |

^a^Complete rate: ‘The number of times the subject actually completed the program’ divided by ‘The number of completion of the program requested in training’

^b^The number of times the subject actually completed the program
